# Lactate dehydrogenase to albumin ratio as an independent factor for 28-day mortality of neonatal sepsis

**DOI:** 10.1038/s41598-025-89108-8

**Published:** 2025-04-30

**Authors:** Xiaohong Xia, Shengfeng Qiu, Xiangjun Cheng, Mengxiao Xie, Jun Zhou

**Affiliations:** 1 Department of Laboratory Medicine, The First Affiliated Hospital of Nanjing Medical University, Nanjing, Jiangsu China; 2 Branch of National Clinical Research Center for Laboratory Medicine, Nanjing, Jiangsu China

**Keywords:** Albumin, 28-day mortality, Neonatal sepsis, Lactate dehydrogenase

## Abstract

Neonatal sepsis significantly contributes to infant mortality rates. The absence of predictive markers has hindered timely clinical intervention, leading to elevated mortality. This study aimed to assess the prognostic relevance of the lactate dehydrogenase (LDH)-to-albumin ratio (LAR) in neonatal sepsis. A retrospective examination was conducted on a cohort of 130 neonates diagnosed with sepsis. Admission laboratory data were gathered. The optimal threshold for LAR was established using receiver operating characteristic curve analysis. Both univariate and multivariate analyses were performed to gauge the predictive efficacy of LAR. A statistical disparity in LAR was noted between survivors and non-survivors (*p* < 0.001). Multivariate analysis confirmed that LAR serves as an independent risk determinant for neonatal sepsis (Hazard ratio [HR] 11.236, 95% Confidence interval [CI] 3.311–38.462, *p* < 0.001). ROC analysis indicated that the area under the curve for LAR was 0.806 across the entire cohort, 0.842 for early-onset sepsis, and 0.737 for late-onset sepsis. Moreover, with a cutoff value set at 23.72, LAR exhibited a prediction specificity of 88.2% and sensitivity of 70.0%. Our research indicates that elevated admission LAR is a negative prognostic indicator in neonatal sepsis, suggesting its potential as a valuable biomarker in clinical settings.

**Supplementary Information:**

The online version contains supplementary material available at 10.1038/s41598-025-89108-8.

## Introduction

Neonatal sepsis is characterized as a clinical syndrome primarily driven by infection, leading to significant morbidity and mortality in newborns^1^. According to epidemiological data spanning the last two decades, the global incidence of neonatal sepsis ranges from 1099 to 4360 cases per 100,000 live births (LBs), with a mortality rate between 11% and 19%^2,3^. In China, a regional study based on birth population estimates the incidence of neonatal sepsis at 25.6 per 1000 LBs^4^. It is important to note that there is considerable variability in neonatal sepsis-related mortality rates across different regions in China^5^. The clinical presentation of sepsis in newborns often includes feeding intolerance, temperature instability, respiratory distress, and lethargy^6,7^, which are inapparent and non-specific symptoms but can quickly develop to disseminated intravascular coagulation, septic shock, and death. Hence, it is crucial to anticipate a poor clinical outcome.

Several investigations have highlighted that elevated serum lactate dehydrogenase (LDH) levels can serve as a prognostic marker for severe adult sepsis^8,9^. Serum albumin has also been utilized as a prognostic indicator for infection, as its levels decline with the worsening of the infection^10^. The LDH to albumin ratio (LAR) is becoming a new inflammatory biomarker. Additionally, previous studies on the LAR have mainly focused on malignant tumors^11^ and severe infection in adult patients^12^. Recent study suggests that LAR was associated with all-cause mortality for adult patients with sepsis in the ICU^13^. Despite increasing interest in the LAR as a prognostic indicator, the utility of LAR in predicting the outcomes of neonatal sepsis remains unexplored. In this study, we aim to assess the value of LAR in forecasting 28-day mortality among neonates with sepsis.

## Material and methods

### Study population

In this retrospective study, we gathered data from 130 neonates who were admitted to the Neonatal Intensive Care Unit (NICU) at Jiangsu Women and Children Health Hospital between January 2014 and November 2019. The study population included any infants diagnosed with sepsis within the first 28 days of life at admission. Neonatal sepsis was identified based on clinical symptoms suggestive of suspected sepsis and fulfillment of at least one of the following criteria: (1) Presence of at least two positive screening parameter results (abnormal C-reactive protein, white blood cell, platelet, erythrocyte sedimentation rate, absolute neutrophil count, or immature to total neutrophil ratio) (Supplementary Table 1 for details); (2) isolation of pathogens from blood or cerebrospinal fluid (the contaminated positive cultures were excluded)^14^. Infants without a follow-up result or with incomplete data were excluded from the study. The study protocol was approved by the Ethics Committee of the First Affiliated Hospital of Nanjing Medical University (2024-SR-136). This study protocol complied with the Declaration of Helsinki. Due to the retrospective nature of the study, the Ethics Committee of the First Affiliated Hospital of Nanjing Medical University waived the need of obtaining informed consent.

### Data collection

Upon admission, demographic details such as gender, age, gestational age at birth (GA), the birth weight, and Apgar scores at 1 and 5 min were documented. For patients diagnosed with sepsis, admission data such as albumin, LDH, routine complete blood count (CBC) of peripheral blood and comprehensive assessments of liver and kidney functions were collected. The study participants were classified into survivor and non-survivor groups based on the primary outcomes, distinguishing between early-onset sepsis (EOS) and late-onset sepsis (LOS). EOS was characterized by sepsis occurring within 3 days post-birth, whereas LOS encompassed sepsis that developed ≥ 3 days after birth. The primary outcome, referred to as 28-day mortality, was established by determining survival status within 28 days from the time of sepsis confirmation.

### Statistical analysis

Variables were presented as mean ± standard deviation or median (range) or frequencies and percentages, and analyzed using Student’s t-test or Mann–Whitney U test, Chi-squared tests. ROC curve analysis was employed for various variables to determine their area under the receiver operating characteristic (AUC) and optimal cut-off values. The optimal cut-off values were used to convert continuous variables with *p* < 0.05 into binary variables. Following univariable analysis (binary variables), multivariate regression analysis (Enter and Forward: Likelihood Ratio (LR)) was conducted to pinpoint independent prognostic factors. The cumulative survival rates of patients with low and high LAR groups were compared using Kaplan–Meier survival curves. The log- rank test was performed to analyze the differences in survival rates between the two groups. Delong test was used to compared the AUCs. All statistical evaluations were carried out using SPSS21 software (SPSS Inc, Chicago, IL, USA). A *p*-value of less than 0.05 was deemed statistically significant.

## Results

### Study cohort

A total of 130 patients were enrolled in this study, with survivors comprising 84.6% (110/130) and non-survivors making up 15.4% (20/130). The positive blood culture rate was 6.4%, and the median age at admission to the NICU was 4 (1, 17) days. The median length of hospital stay was 16 (11, 27) days, while in the mortality group, the median age at death was 5 (3, 15) days. The study included 77 male patients and 53 female patients. No significant gender differences, median age, or weight were noted between the survivor and non-survivor groups; however, there were substantial variations in gestational age at birth and Apgar scores. Among the laboratory findings, platelet, aspartate aminotransferase (AST), LDH, albumin (ALB), albumin to globulin ratio (A/G), and creatinine exhibited significant differences between non-survivors and survivors. Additionally, LAR was notably higher in the non-survivor group (30.42 ± 15.25 vs. 17.12 ± 7.64, *p* < 0.001) (Table 1).

**Table 1:** Baseline characteristics of study population.

Variables	Non-survivor (N = 20)	Survivor (N = 110)	*p*-value
Gender (male/female), n	12/8	65/45	0.940
Age (day)	2 (0.5, 28)	6(0.1, 28)	0.124
Gestational age at birth (weeks)	33 (28, 37)	36 (35, 36)	**0.008**
Birth weight (g)	2100 (1175, 2387)	2500 (1600, 2689)	0.055
Apgar score (1 min)	7 (4, 10)	10 (8, 10)	** < 0.001**
Apgar score (5 min)	8 (5, 10)	10 (8, 10)	** < 0.001**
CRP (mg/L)	4 (1, 58)	5 (1, 104)	0.448
WBC (× 10^9^/L)	11.05 (5.02, 105.13)	11.38 (2.21, 61.79)	0.096
Lymphocyte (× 10^9^/L)	6.26 ± 6.19	4.77 ± 2.78	0.083
Neutrophil (× 10^9^/L)	5.71 (1.44, 78.64)	5.01 (0.37, 51.35)	0.153
Hemoglobin (g/L)	156 ± 29	164 ± 28	0.222
Platelet (× 10^9^/L)	201 ± 76	249 ± 104	**0.049**
ALT (U/L)	9.6 ± 5.7	12.6 ± 10.4	0.199
AST (U/L)	65.2 ± 30.2	49.9 ± 30.9	**0.043**
LDH (U/L)	779.6 ± 348.4	558.0 ± 252.0	**0.001**
Albumin (g/L)	27.0 ± 7.2	33.0 ± 4.4	** < 0.001**
A/G	1.9 ± 0.7	2.2 ± 0.5	**0.032**
Urea (mmol/L)	5.1 ± 2.9	4.5 ± 6.2	0.696
Creatinine (umol/L)	62.1 ± 22.2	54.7 ± 43.7	**0.045**
LAR	30.42 ± 15.25	17.12 ± 7.64	** < 0.001**

CRP: C-reactive protein; WBC: white blood count; ALT: alanine aminotransferase; AST: aspartate transaminase; LDH: lactate dehydrogenase; A/G: albumin to globulin ratio; LAR: lactate dehydrogenase to albumin ratio. Data are median (range) or mean ± SD. Bold values indicate statistical significance (*p* < 0.05).

### Association between laboratory values and mortality

Univariate regression analysis revealed that platelet, AST, LDH, ALB, A/G, creatinine, and LAR all demonstrated statistical significance between the survivor and non-survivor groups (all *p* < 0.05). Subsequently, the aforementioned indicators (platelet, AST, LDH, ALB, A/G, creatinine, and LAR) were converted into binary variables and incorporated into the multivariate analysis (Enter and Forward: Likelihood Ratio (LR)), the results identified that only ALB (Hazard ratio [HR] = 9.066, 95% confidence interval [CI] 2.433–33.775, *p* = 0.001) and LAR (HR = 11.236, 95% CI: 3.311–38.462, *p* < 0.001) as independent risk factors for mortality in neonatal sepsis (Table 2).

**Table 2:** Univariate and multivariate analyses of factors which affect 28-day mortality.

Variables	Cut-off value	Univariate analyses			Multivariate analyses		
		HR	95% Cl	*p*-value	HR	95% Cl	*p*-value
Platelet	210 × 10^9^/L	3.250	1.198–8.813	0.025			
AST	45.7 U/L	3.774	1.348–10.638	0.013			
LDH	625.5 U/L	5.714	2.066–15.873	0.001			
Albumin	28.4 g/L	9.500	3.333–27.074	< 0.001	9.066	2.433–33.775	0.001
A/G	2	3.078	1.100–8.611	0.031			
creatinine	51.3 umol/L	3.731	1.269–10.989	0.015			
LAR	23.72	17.544	5.682–52.632	< 0.001	11.236	3.311–38.462	< 0.001

AST: aspartate transaminase; LDH: lactate dehydrogenase; A/G: albumin to globulin ratio; LAR: lactate dehydrogenase to albumin ratio; CI: Confidence interval; HR: Hazard ratio.

#### Predictive accuracy for mortality

**Fig. 1:**
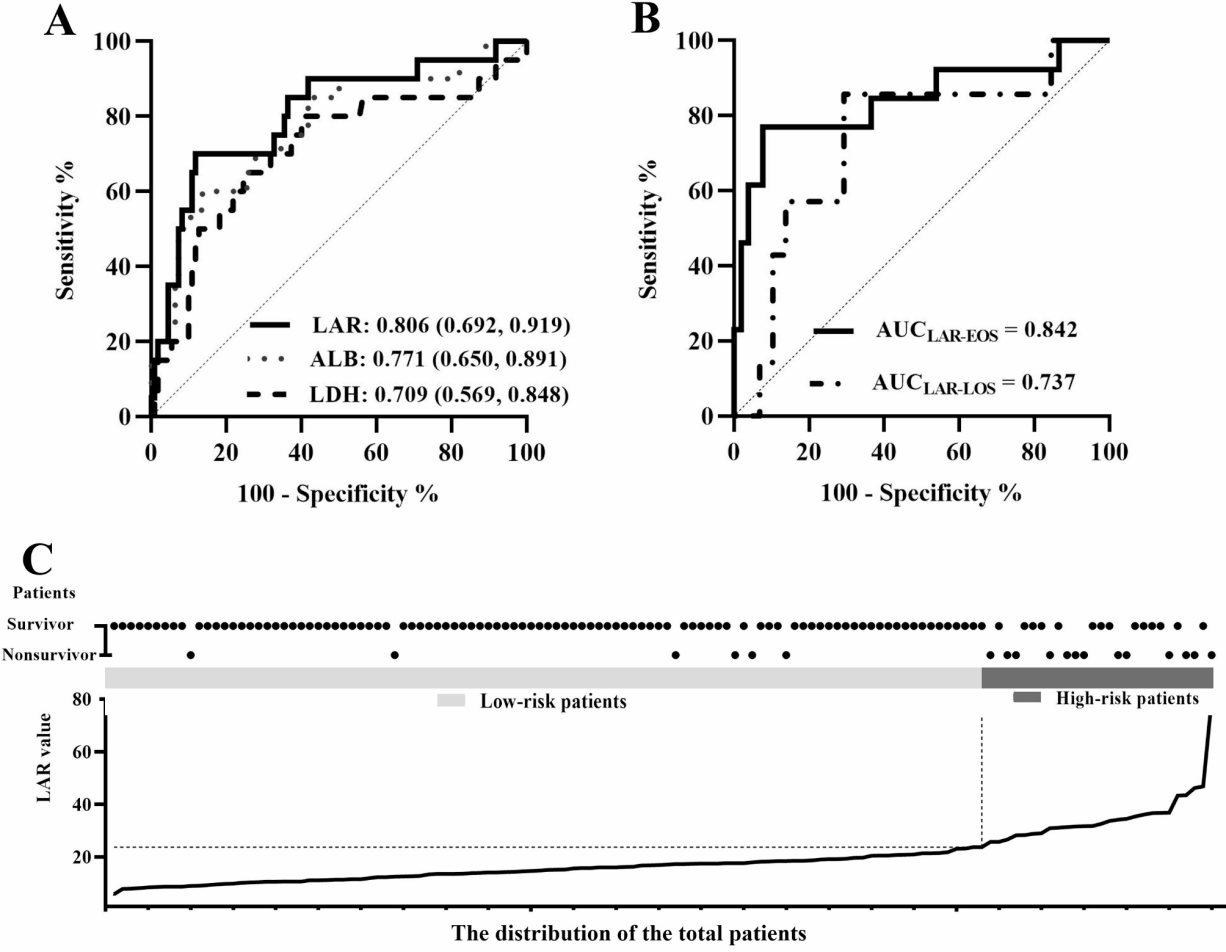
Performance of LDH, ALB and LAR. (**A**) AUC of LDH, ALB and LAR for predicting mortality in total patients. (**B**) AUC of LAR for predicting mortality in the subgroup patients. (**C**) The distribution of the total patients.

ROC curve analysis was conducted to evaluate the predictive value of LDH, ALB, and LAR for neonatal sepsis mortality. Figure 1A illustrates that the AUCs for LDH, ALB, and LAR were 0.709, 0.771, and 0.806, respectively. The optimal cut-off value for LAR was determined to be 23.72, with a specificity of 88.2% and a sensitivity of 70.0% (Supplementary Table 2). Figure 1C displays the distribution of mortality among the participants.

**Fig. 2:**
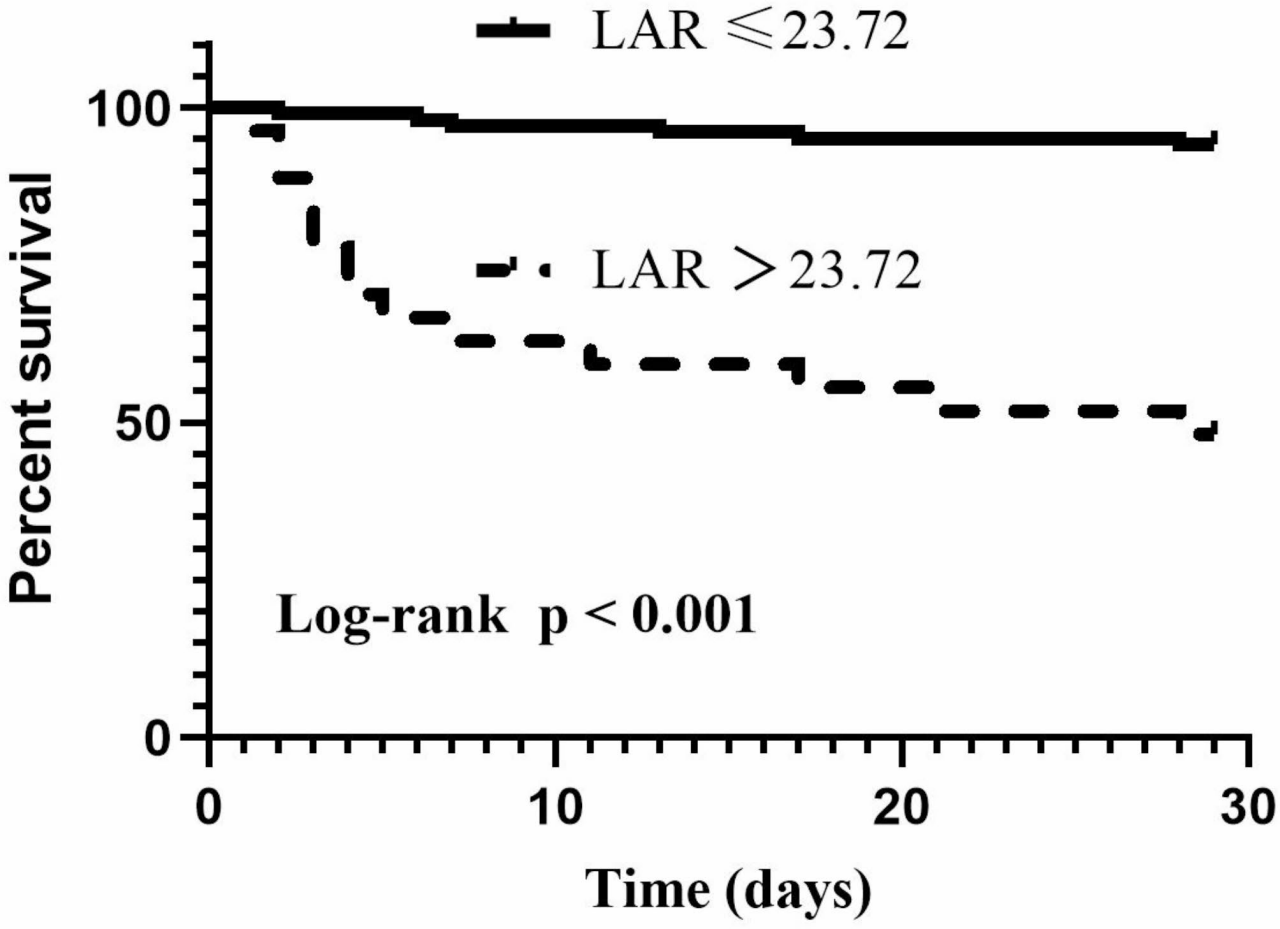
Kaplan–Meier survival curves for cumulative survival rates in the low and high LAR groups.

Kaplan Meier survival curves were constructed to evaluate the cumulative survival rates of different LAR levels, revealed that neonates suffering from sepsis had a significantly lower survival rate with LAR > 23.72 (*p* < 0.001) (Fig. 2).

A total of 130 cases of neonatal sepsis, comprising 65 cases of early-onset sepsis (EOS) and 65 cases of late-onset sepsis (LOS), resulting in a ratio of 1:1 between the two groups. Furthermore, patients were categorized into the EOS and LOS groups. ROC analysis indicated that the AUC for LAR was 0.842 for EOS and 0.737 for LOS (Fig. 1B).

## Discussion

In our study, we found that LAR levels were significantly higher in the non-survivor group. Furthermore, LAR emerged as an independent risk factor for 28-day mortality in neonatal sepsis. The ROC curve analysis demonstrated that the AUC for LAR in predicting neonatal sepsis mortality was superior to that of other variables. With the optimal cut-off value of 23.72 for LAR, the specificity reached 88.2%, while the sensitivity was 70.0%. Elevated LAR (> 23.72) was associated with high-risk neonates suffering from sepsis.

LDH, an essential enzyme in cellular metabolism found in nearly all living cells, is released during tissue damage and involved in multiple pathophysiological processes. Notably, LDH serves as a non-specific marker of cell death in numerous diseases. Numerous studies have linked elevated serum LDH levels to conditions such as infection, acute myocardial infarction, sepsis, malignancies, and COVID-19^15–17^. However, due to its elevation in various diseases, LDH is not typically regarded as a specific prognostic indicator.

ALB, considered a negative acute-phase protein in inflammation, has been extensively studied for its close association with inflammatory states^18,19^. Inflammatory conditions can reduce albumin production by the liver through increased interleukin-1 or tumor necrosis factor^20^. Yang et al.^10^ noted that neonates with sepsis often exhibit hypoalbuminemia, and lower albumin levels may correlate with a poorer prognosis or more severe inflammation. In addition to being used as an individual prognostic marker, albumin is also combined with other variables to improve the prognostic value of sepsis. The lactate to albumin ratio was a useful prognostic factor for critically ill sepsis patients^21^. C-reactive protein-to-albumin ratio (CAR) was an independent predictor for the severity of neonatal sepsis^22^.

Both LDH and ALB are readily accessible in routine clinical settings and are linked to severe infections^8^. LAR combines factors of inflammation, nutritional status and chronic disease, may provide more comprehensive prognostic value than individual predictive values of LDH or ALB. Previous research has indicated that elevated LAR levels are associated with a poor prognosis and increased mortality. Aday et al.^23^ assessed 295 patients with colorectal carcinoma post-curative resection and found that LAR ≥ 52.7 g/dL significantly correlated with worse disease-free survival and overall survival. Komac et al.^24^ suggested that LAR could be a valuable inflammatory marker for adult-onset Still’s disease patients with organ involvement and high ferritin levels. Yan et al.^25^ reported that patients with higher LAR (> 6.75) had nearly three times the risk of stroke-associated pneumonia (SAP), with the risk of SAP rapidly increasing as LAR values rose between 6 and 10. It is noteworthy that our study further supports this point that higher LAR (≥ 23.72) refers to a poor prognosis in neonatal sepsis, emphasizing the necessity of increasing clinical vigilance.

In addition, in our previous study, we found that neutrophil- to- monocyte ratio (NMR) was a promising prognostic factor for neonatal sepsis^26^. When compared it with LAR in this study, LAR exhibited better predictive ability in the prognosis of neonatal sepsis.

This study, however, had several limitations. Firstly, it was a retrospective analysis, which may introduce selection bias, necessitating further prospective studies to validate its applicability. Secondly, being a single-center study, broader validation is required to ascertain the generalizability of these findings. Lastly, we only collected initial LAR levels upon admission, and considering dynamic monitoring of LAR throughout hospitalization could provide additional insights.

In conclusion, elevated LAR levels were associated with a poor prognosis in neonatal sepsis. LAR may serve as a useful independent prognostic factor for neonatal sepsis.

## Electronic supplementary material

Below is the link to the electronic supplementary material.

Supplementary Material 1

Supplementary Material 2

## Data Availability

The data and materials can be found from the corresponding author.
