# Supplementary material for: Lactate dehydrogenase to albumin ratio as an independent factor for 28-day mortality of neonatal sepsis
Source: Sci Rep. 2025 Apr 30;15:15158. doi: 10.1038/s41598-025-89108-8 (PMC12043797; doi:10.1038/s41598-025-89108-8)
Supplement: Supplementary file 1 — Supplementary Material 1 [file 41598_2025_89108_MOESM1_ESM.docx]

**Supplementary Table 1. The abnormal ranges of diagnostic parameters for neonatal sepsis**

| **Diagnostic Parameter** | **Abnormal Range** |
| --- | --- |
| **White blood cell** | ≥25×10⁹/L in the first 3 days after birth, or ≥20×10⁹/L after 3 days, or <5×10⁹/L at any age |
| **C-reactive protein** | ≥3mg/L in the 6 hours after birth, or ≥5mg/L within 6-24 hours after birth, or ≥10mg/L after 24 hours after birth |
| **Platelet** | <100×10⁹/L |
| **Absolute neutrophil count** | <1×10⁹/L |
| **Immature to total neutrophil ratio** | ≥0.16 in the first 3 days after birth, or ≥0.12 after 3 days |
| **Procalcitonin** | ≥0.5μg/L within 6 hours after birth or after 72 hours |
| **Erythrocyte sedimentation rate** | ≥21mm/h for male, ≥26mm/h for female |
