# Supplementary material for: Lactate dehydrogenase to albumin ratio as an independent factor for 28-day mortality of neonatal sepsis
Source: Sci Rep. 2025 Apr 30;15:15158. doi: 10.1038/s41598-025-89108-8 (PMC12043797; doi:10.1038/s41598-025-89108-8)
Supplement: Supplementary file 2 — Supplementary Material 2 [file 41598_2025_89108_MOESM2_ESM.doc]

**Supplementary Table 2. Performance for clinical predication model**

| **Factors** | **Model fixed at maximum Youden’s Index*** | | | | **P_1_ value** | **P_2_ value** |
| --- | --- | --- | --- | --- | --- | --- |
|  | **Cut-off value** | **AUC (95% CI)** | **Specificity** | **Sensitivity** |  |  |
| **LDH** | 625.5 | 0.709 (0.569-0.848) | 75.5% | 65.0% | Reference | 0.510 |
| **ALB** | 28.4 | 0.771 (0.650-0.891) | 86.4% | 60.0% | 0.510 | Reference |
| **LAR** | 23.72 | 0.806 (0.692-0.919) | 88.2% | 70.0% | 0.042 | 0.628 |

LDH: lactate dehydrogenase; ALB: albumin; LAR: lactate dehydrogenase to albumin ratio; AUC: Area under the receiver operator characteristic curve; CI: Confidence interval.

Youden’s index=Sensitivity+ Specificity- 1.

Delong test was used to compared the AUCs.
